# Magnesium enhances aurintricarboxylic acid’s inhibitory action on the plasma membrane Ca^2+^-ATPase

**DOI:** 10.1038/s41598-024-65465-8

**Published:** 2024-06-26

**Authors:** Cecilia A. Souto-Guevara, Diego Obiol, Camila L. Bruno, Mariela S. Ferreira-Gomes, Juan Pablo F. C. Rossi, Marcelo D. Costabel, Irene C. Mangialavori

**Affiliations:** 1 Universidad de Buenos Aires, Facultad de Farmacia y Bioquímica, Consejo Nacional de Investigaciones Científicas y Técnicas (CONICET), Instituto de Química y Fisicoquímica Biológicas Dr. Alejandro Paladini (IQUIFIB), Junín 956, C1113AAD Buenos Aires, Argentina; 2 Departamento de Física, Instituto de Física del Sur (IFISUR), Universidad Nacional del Sur (UNS), CONICET, B8000CPB Bahía Blanca, Argentina

**Keywords:** PMCA, Plasma membrane Ca^2+^-ATPase, ATA, Inhibition mechanism, ATA-magnesium complex, ATA-binding site, Biochemistry, Biophysics

## Abstract

Our research aimed to elucidate the mechanism by which aurintricarboxylic acid (ATA) inhibits plasma membrane Ca^2+^-ATPase (PMCA), a crucial enzyme responsible for calcium transport. Given the pivotal role of PMCA in cellular calcium homeostasis, understanding how it is inhibited by ATA holds significant implications for potentially regulating physiopathological cellular processes in which this pump is involved. Our experimental findings revealed that ATA employs multiple modes of action to inhibit PMCA activity, which are influenced by ATP but also by the presence of calcium and magnesium ions. Specifically, magnesium appears to enhance this inhibitory effect. Our experimental and in-silico results suggest that, unlike those reported in other proteins, ATA complexed with magnesium (ATA·Mg) is the molecule that inhibits PMCA. In summary, our study presents a novel perspective and establishes a solid foundation for future research efforts aimed at the development of new pharmacological molecules both for PMCA and other proteins.

## Introduction

Aurintricarboxylic acid (ATA) is a versatile compound that exhibits a wide range of effects on cellular processes involving enzymes and proteins. This biochemical agent has garnered significant interest due to its diverse functionality, which can be categorized into key areas like enzymatic inhibition. ATA demonstrates potent inhibitory capabilities against various enzymes crucial for cellular function^1–3^. It can inhibit ATPases, which are enzymes responsible for energy transfer within cells, as well as ribonucleases involved in RNA processing^4,5^. This inhibition disrupts normal cellular activities, making ATA a valuable tool for studying the roles of these enzymes. Extensive research has unveiled the promising antiviral potential of ATA. Studies have shown that ATA can effectively combat viral replication in viruses such as coronavirus (including SARS-CoV-2)^6–8^ and influenza^9,10^. Its mode of action involves interfering with the virus’s ability to replicate, thereby hampering its propagation within host cells. However, further investigations are necessary to establish ATA’s suitability as an antiviral therapeutic agent for human use. In addition to its enzymatic inhibitory effects, ATA has been observed to interact with various proteins^7,8,11^. The precise mechanisms underlying these interactions and their subsequent impact on cellular processes are currently under investigation. Nonetheless, the multifaceted nature of ATA's effects makes it an intriguing compound for further exploration in the fields of biochemistry, pharmacology, and medicine.

The plasma membrane Ca^2+^-ATPase (PMCA) plays a crucial role in maintaining calcium ion balance across cell membranes^12^. It actively transports calcium ions out of the cell, contributing to cellular homeostasis. PMCA belongs to the P-ATPases, a family of proteins that utilize the energy derived from ATP hydrolysis to transport ions and molecules across biological membranes^13^. Like all P-ATPases, PMCA follows the E1–E2 model and undergoes various conformational changes as part of its reaction cycle to carry out its transport function^14^. This model proposes that cytoplasmic Ca^2+^ binds with high affinity to *E*1 state, forming *E*1Ca, which is then phosphorylated by ATP, leading to *E*1(Ca)P. In *E*1(Ca)P state, Ca^2+^ is occluded and inaccessible to both sides of the membrane. A conformational change leads to the *E*2P state, which has low affinity for Ca^2+^ and it is released to the extracellular medium. Dephosphorylation of *E*2P leads to *E*2, which in the presence of Ca^2+^, transitions back to the *E*1 state to initiate another transport cycle. These intermediate states of the PMCA reaction cycle have different characteristics and can be studied using ligands that stabilize or “fix” their conformation.

**Figure 1:**
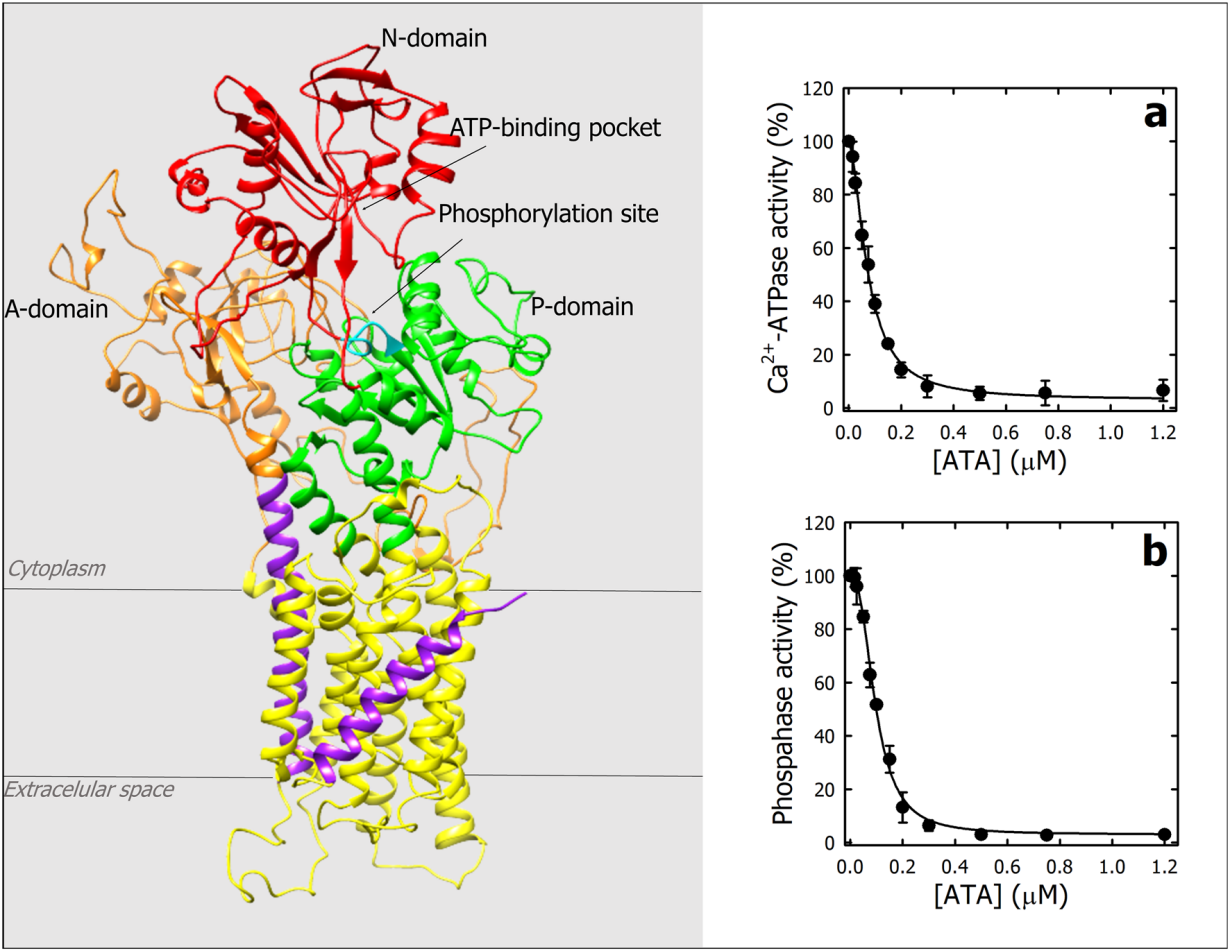
Effect of ATA on Ca^⁠2+^‑ATPase and phosphatase activities of PMCA. (**a**) Cryo-EM structure of PMCA1^16^. The N-, A- and P-domains are shown in red, orange and green, respectively. The nucleotide-binding pocket and the phosphorylation site (Asp475) are qualitatively indicated with black arrows. In the transmembrane domain, M1 and M10 are colored in light violet and M2–M9 are colored in yellow. Ca^⁠2+^‑ATPase (**b**) and phosphatase (**c**) activities of PMCA as a function of ATA concentration were determined as described in “Methods” section. In both Panels, the activity of PMCA in the absence of ATA was taken as 100%. The continuous lines represent the fitting of Eq. (1) to experimental data. The values obtained for the parameter were: (a) *K*_*i*_ = 0.073 ± 0.003 µM; *v*_*∞*_ = 3.0 ± 1.6% and *n* = 1.8 ± 0.1 and, (b) *K*_*i*_ = 0.097 ± 0.004 µM; *v*_*∞*_ = 2.6 ± 0.6%, and *n* = 2.5 ± 0.2. Values are the mean ± SE of three independent experiments.

In humans, there are four isoforms of PMCA (PMCA1 to 4). Isoforms 1 and 4 are ubiquitously distributed, while isoforms 2 and 3 are restricted to specialized tissues^15^. The hydrophobic domain of PMCA is made up of 10 transmembrane segments (M1 to 10) while the cytoplasmic region are the A (actuator), P (phosphorylation) and N (nucleotide binding) domains, characteristic of P-ATPases^16^ (Fig. 1a). Interestingly, Mohamed et al. evaluate a library of medically optimized drug-like molecules and propose that ATA specifically inhibits PMCA4, leading to perturbations in intracellular calcium levels^17^. ATA was utilized to investigate the involvement of PMCA4 in endothelial cell motility and blood vessel formation regulated by calcineurin/NFAT signaling^18^. While the cellular-level studies yielded promising results, the utilization of ATA in in-vivo models revealed significant toxicity^18^. Consequently, to further explore the potential therapeutic implications of ATA on PMCA, including improved and more specific versions, it becomes imperative to first address certain critical aspects that have not been explored, such as:

1. The precise mechanisms by which ATA inhibits PMCA function are not fully understood. Unraveling these mechanisms could provide valuable insights into cellular calcium regulation.
2. How ATA interacts with calcium (Ca^2+^) and magnesium (Mg^2+^) ions within the context of PMCA inhibition warrants further investigation. Does ATA directly compete with these ions for the binding sites?
3. Effect of ATA on the ATP binding to PMCA is another intriguing area. Does ATA alter ATP- binding affinity or disrupt ATP-dependent processes?

In summary, while the inhibitory effect of ATA on PMCA has been established^17,18^, deeper exploration is necessary to elucidate the underlying mechanisms and interactions. Here, we present a systematic study characterizing the mechanism of PMCA inhibition by ATA through steady-state and equilibrium experiments, complemented by flexible molecular docking studies. This groundwork lays the foundation for future research endeavors in this field, as our findings carry significant implications for understanding and potentially treating disorders associated with PMCA dysfunction.

## Results and discussion

### Effect of ATA on the PMCA activity

Figure 1b shows PMCA Ca^2+^-ATPase activity as a function of increasing ATA concentrations. The continuous line corresponds to the fitting of Eq. (1) to the experimental data,

1 $$v =\frac{{v}_{0-}{v}_{\infty }}{1+{([ATA]/{K}_{i})}^{n}},$$

where *v*_*0*_ and *v*_*∞*_ correspond to the PMCA Ca^2+^-ATPase activity in the absence of ATA and when its concentration tends to infinity, *K*_*i*_ is the ATA concentration at which half the maximum effect is observed, and* n* is the Hill coefficient. The values obtained for these parameters were 0.073 ± 0.003 µM; *v*_*∞*_ = 3.0 ± 1.6% and *n* = 1.8 ± 0.1.

The PII-ATPases can hydrolyze other substrates in the absence of the transported ion, indicating that this phosphatase activity is not linked to ion transport. PMCA hydrolyzes p-nitrophenyl phosphate (pNPP), and this phosphatase activity is inhibited by Ca^2+^, suggesting its association with the *E*2 conformation^19^. Figure 1c shows the phosphatase activity of PMCA as a function of the ATA concentration. The continuous line represents the fitting of Eq. (1) to the experimental data, but in this case, *v*_*0*_ and *v*_*∞*_ refer to the PMCA phosphatase activity. The values obtained for these parameters were: *K*_*i*_ = 0.097 ± 0.004 µM; *v*_*∞*_ = 2.6 ± 0.6%, and *n* = 2.5 ± 0.2. These results indicate that ATA inhibits both the Ca^2+^-ATPase and phosphatase activities of PMCA isolated from human erythrocytes with high apparent affinity.

### Effect of calcium, magnesium, and ATP on inhibition of PMCA by ATA

**Figure 2:**
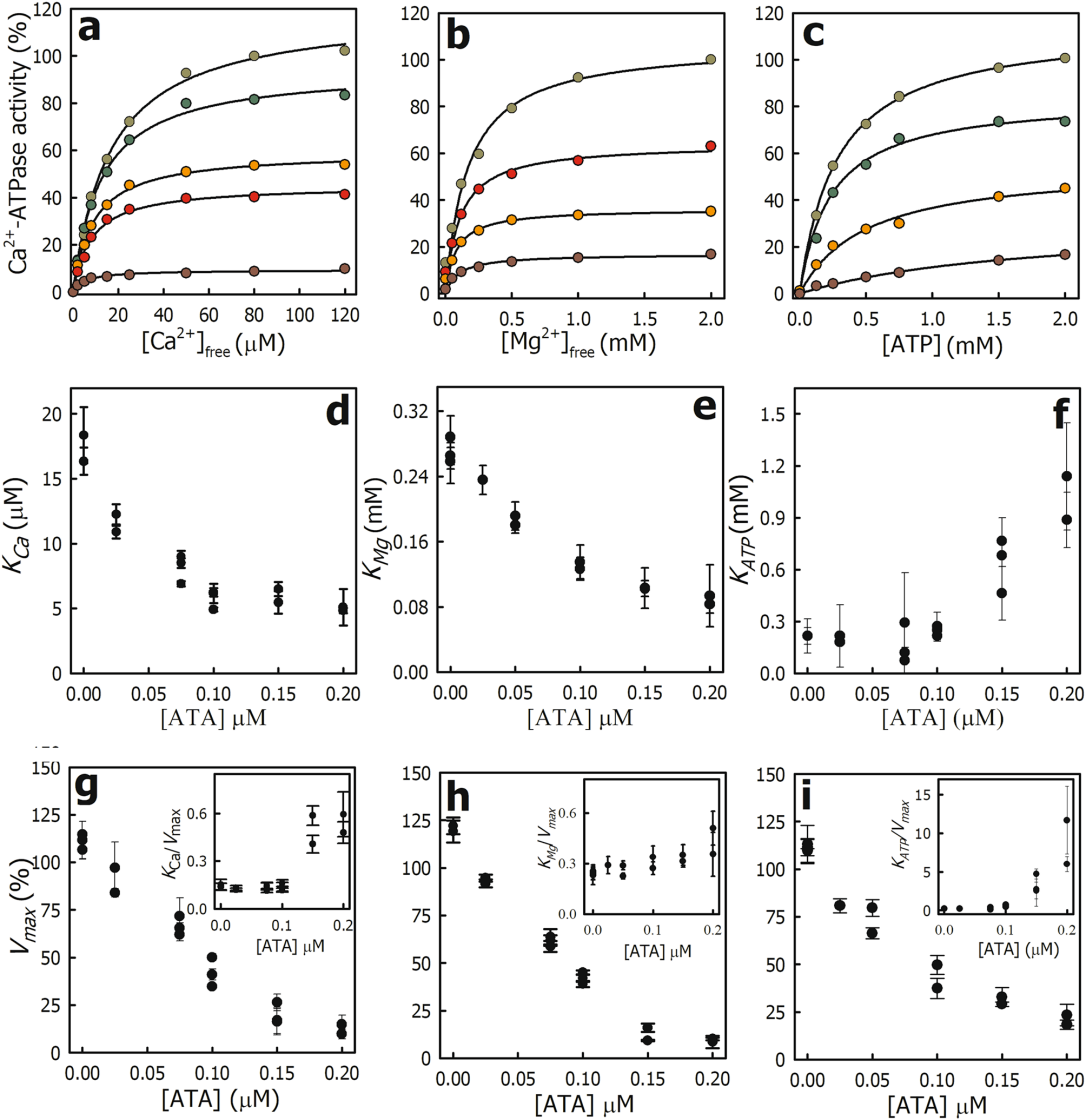
Effect of ATA on the apparent affinity of PMCA for Ca^2+^, Mg^2+^ and ATP. (**a–c**). PMCA Ca^2+^-ATPase activity as a function of the concentration of free Ca^2+^ (**a**); free Mg^2+^ (**b**) or ATP·Mg (**c**) was determined in the presence of different ATA concentrations (*from top to bottom*: in (**a**) 0.0; 0.025; 0.075; 0.100 and, 0.200 µM ATA; in (**b,c**) 0.0; 0.050; 0.100 and, 0.150 µM ATA). In each Panel, the maximal Ca^2+^-ATPase activity obtained in the absence of ATA was normalized to 100%. The continuous lines in A-C represent the fitting of Eq. (5) to the experimental data. (**d–i**) The values obtained for the PMCA apparent affinity for Ca^2+^ (*K*_*Ca*_); Mg^2+^ (*K*_*Mg*_) and ATP (*K*_*ATP*_) (**d–f**) as well as the maximal velocity (**g–i**) were plotted as a function of ATA concentration. *Inset* in panels (**g–i**) show the *K*_*x*_*/V*_*max*_ ratio for each case. Panels (**a–c**) showcase representative experiments, while panels (**d–i**) present values derived from three to four independent experiments.

Figure 2a shows the Ca^2+^-ATPase activity of PMCA as a function of free Ca^2+^ in the presence of different ATA concentrations. In all cases, the experimental data were described by Eq. (5). Table 1 shows the parameter obtained in the absence and presence of 0.15 µM ATA. When ATA concentrations increased, *K*_*Ca*_ (Fig. 2d) and *V*_*max*_ decreased (Fig. 2g), and the *K*_*Ca*_/*V*_*max*_ ratio was not constant (*inset* in Fig. 2g). These results suggest that in PMCA, ATA behaves as a mixed-type inhibitor with respect to Ca^2+^. In this mechanism, the inhibitor binds with different affinity to the free enzyme or the enzyme–substrate complex, meaning that the presence of the substrate modifies the affinity for the inhibitor and vice versa^20^. This agrees with the fact that ATA also inhibits the phosphatase activity of PMCA with apparent high affinity (Fig. 1c).

**Table 1:** Effect of ATA on V_max_ and apparent affinity (K_x_) of PMCA for Ca^2+^, Mg^2+^ and ATP.

*x*	*V*_*max*_ (%)		*K*_*x*_ (µM)	
	–	ATA*	–	ATA*
Ca^2+^	119.4 ± 5.9	16.0 ± 2.2	16.4 ± 1.4	6.5 ± 0.5
Mg^2+^	113.2 ± 2.4	29.1 ± 1.2	288.1 ± 26.9	102.2 ± 9.8
ATP	114.7 ± 1.0	29.1 ± 1.2	215.9 ± 6.8	765.9 ± 16.0

The parameters were derived by fitting Eq. (5) to the experimental data shown in Fig. 2a–c.

The values marked with an asterisk (*) were obtained in the presence of 0.15 μM aurintricarboxylic acid (ATA), which is approximately 2 times the *K*_*i*_ value determined in Fig. 1b.

Like other members of the P-ATPase family, Mg^2+^ is an essential cofactor for PMCA. On one hand, the true substrate is ATP·Mg, and on the other, PMCA possesses a binding site for Mg^2+^. Figure 2b shows the PMCA Ca^2+^-ATPase activity as a function of free Mg^2+^ in the presence of different ATA concentrations. The continuous lines correspond to the fitting of Eq. (5) to the experimental data. In the presence of increasing ATA concentrations, *K*_*Mg*_ (Fig. 2e) and *V*_*max*_ (Fig. 2h) decreased (see also Table 1), and the *K*_*Mg*_ /*V*_*max*_ ratio remained approximately constant (*inset* in Fig. 2h). These results suggest that ATA behaves as an uncompetitive inhibitor with respect to Mg^2+^. In this mechanism, the inhibitor binds to the enzyme–substrate complex, meaning an increase in the apparent affinity for the substrate is observed^20^.

Figure 2c shows the PMCA Ca^2+^-ATPase activity as a function of ATP (ATP·Mg) in the presence of different ATA concentrations. The experimental data were described by Eq. (5) and *K*_*ATP*_ represents an overall value between the PMCA apparent affinity by ATP at the catalytic site (high affinity) and the regulatory site (low affinity)^21^. *K*_*ATP*_ (Fig. 2f) remained approximately constant up to 0.1 µM ATA and then increased, while *V*_*max*_ (Fig. 2i) decreased throughout the range evaluated (see also Table 1). Thus, the *K*_*ATP*_/*V*_*max*_ ratio as a function of ATA concentration was not constant (*inset* in Fig. 2i). These results suggest that in PMCA, ATA behaves as a mixed-type inhibitor with respect to ATP, i.e., ATA does not bind to the ATP-binding site but affects the affinity of PMCA for this nucleotide.

### Interaction of ATA with magnesium

**Figure 3:**
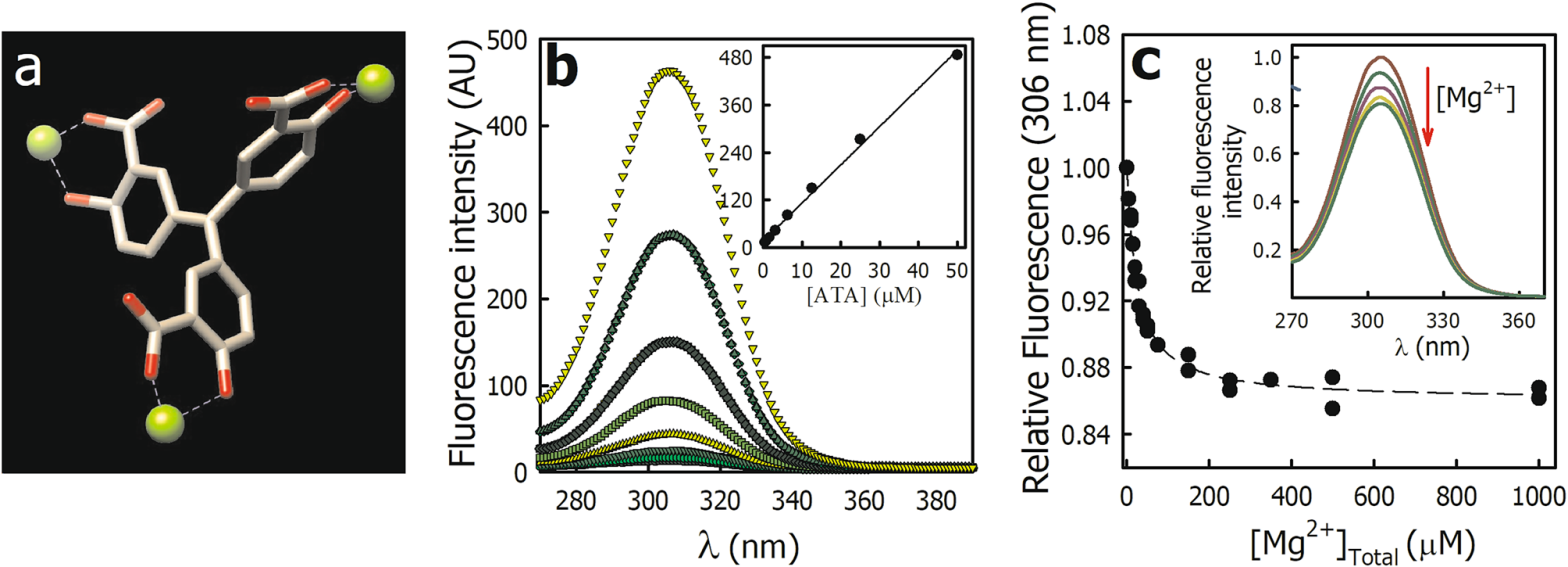
Interaction of ATA and Mg^2+^ evaluated by fluorescence. (**a**) Structure of Triaurinecarboxylic Acid coordinated with Mg^2+^ (ATA·Mg). (**b**) Excitation spectra in the presence of increasing ATA concentrations in the reconstitution medium of PMCA. *Inset*. Fluorescence at 306 nm as a function of ATA concentration (*from top to bottom*: 0.4; 0.8; 1.6; 3.1; 6.3; 12.5; 25 and 50 µM ATA). (**c**) ATA fluorescence at 306 nm as a function of the Mg^2+^ concentration. The λ_em_ was 425 nm and the fluorescence in the absence of Mg^2+^ (1 mM EDTA) was normalized to 1. The discontinuous line represents the fitting of Eq. (2) to the experimental data and the values obtained for *b* and *F*_*∞*_ parameter were 24.1 ± 2.1 µM and 0.860 ± 0.002, respectively*. Inset*. Excitation spectra of ATA in the presence of increasing Mg^2+^ concentrations (*from top to bottom*: 0; 20; 50; 100 and, 1000 µM Mg^2+^). Panel (**b**) and *inset* in (**c**) show representative experiments, Panel (**c**) corresponds to the results obtained in three independent experiments.

The interaction of ATA with metals has been described early and its employ to detect aluminum in water in a standard assay^22^. Besides, ATA can form complexes with alkaline earth metals such as Sr^2+^, Ba^2+^, Ca^2+^, and Mg^2+^ (Fig. 3a)^23^. Thus, the cooperative behavior observed in the inhibition of Ca^2+^-ATPase activity (Fig. 1b) could be because the molecule that binds to PMCA is ATA·Mg (or ATA·Ca^2+^), and this complex forms in low amounts at low ATA concentrations.

Figure 3b shows the fluorescence excitation spectrum of ATA in the reaction medium in which the Ca^2+^-ATPase activity of PMCA was determined, but in the absence of ATP and protein. The excitation maximum was observed at 306 nm, and the fluorescence intensity increased linearly as a function of the ATA concentration (*inset* in Fig. 3b). These results agree with those previously described for aqueous solutions of ATA at pH 7.4^7^ and suggest that the molecule is a monomer at the evaluated concentrations.

Figure 3c shows the fluorescence at 306 nm of 10 µM ATA as a function of the Mg^2+^ concentrations. The addition of Mg^2+^ decreased the ATA fluorescence with a saturable behavior without significant changes in other characteristics of the spectrum *(inset* in Fig. 3c). The experimental data were well described by Eq. (2),

2 $$F={F}_{\infty }+\frac{\left({F}_{0}-{F}_{\infty }\right) \cdot b}{b+[{Mg}^{2+}]},$$

where *F*_0_ and *F*_*∞*_ correspond to the fluorescence in the absence of Mg^2+^ and when its concentration tends to infinity, respectively, and *b* corresponds to the added Mg^2+^ concentration at which half the maximum effect is achieved. The values obtained for *b* and *F*_*∞*_ were 24.1 ± 2.1 µM and 0.860 ± 0.002, respectively. A similar result was obtained in the absence of Mg^2+^ and increasing concentrations of Ca^2+^ (data not shown). These results suggest that in the conditions in which Ca^2+^-ATPase activity was determined (Fig. 1b), most of the ATA would be found as ATA·Mg complex and, consequently, it would be the form that inhibits PMCA. In agreement with this, the phosphatase activity of PMCA, which is determined in the absence of Ca^2+^ and in the presence of Mg^2+^, was inhibited by ATA (Fig. 1c).

### Binding of ATA to PMCA assessed by fluorescence

#### ATA fluorescence in the presence of PMCA

To evaluate the binding of ATA to PMCA, we studied the changes in fluorescence of both molecules in different experimental conditions.

**Figure 4:**
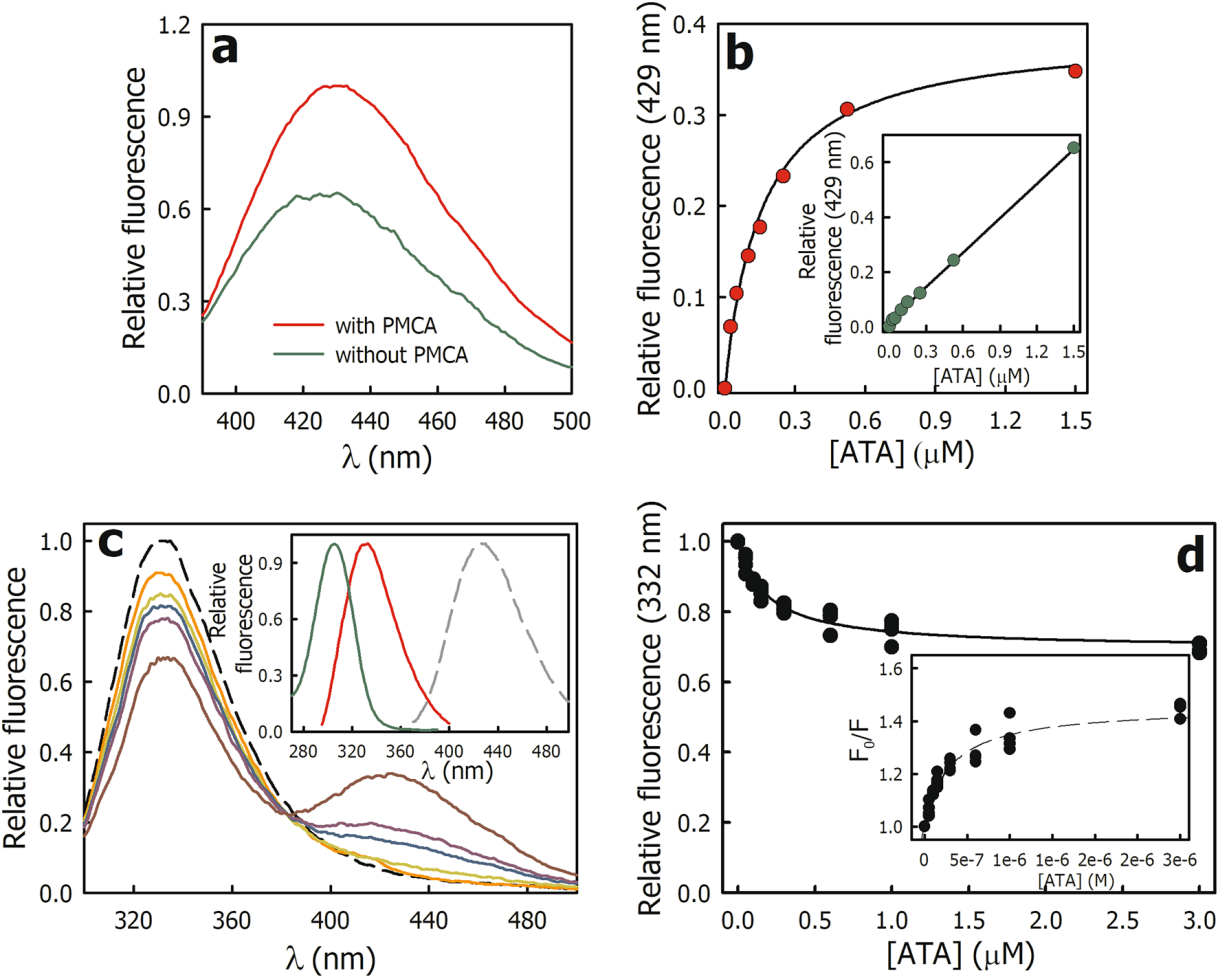
Binding of ATA to PMCA assayed by fluorescence. (**a**) Fluorescence emission spectrum of 1.5 μM ATA in the absence (*green line*) and presence (*red line*) of PMCA. The λ_ex_ was 306 nm and the fluorescence intensity at 429 nm in the presence of PMCA was normalized to 1. (**b**) Relative fluorescence of ATA bound to PMCA at 429 nm as a function of the ATA concentration. The continuous line represents the fitting of Eq. (3) to the experimental data and the value obtained for *Kʹ*_*ATA*_ was 0.16 ± 0.02 µM. *Inset.* Relative fluorescence of ATA at 429 nm as a function of ATA concentration in the absence of PMCA. The continuous line represents the fitting of a linear equation to the experimental data. (**c**) Fluorescence emission of PMCA in the absence (*dashed black line*) and in the presence of increasing concentrations of ATA. The λ_ex_ was 290 nm and the fluorescence intensity at 332 nm in the absence of ATA was normalized to 1. *Inset.* Fluorescence emission spectra of PMCA (*red line*) and ATA (*dashed gray line*), and fluorescence excitation spectrum of ATA (*green line*). (**d**) Intrinsic fluorescence of PMCA at 332 nm as a function of ATA concentration. The continuous line represents the fitting of Eq. (2) to the experimental data and the values obtained for *F*_*min*_ and *Kʹʹ*_*ATA*_ were 0.69 ± 0.09 and 0.19 ± 0.02 µM, respectively. *Inset*. Stern–Volmer plot, where *F*_0_ and *F* are the fluorescence at 332 nm in the absence and presence of different concentrations of ATA (*quencher*), respectively. The dashed line is illustrative to facilitate visualization of the behavior of the experimental data. Panels (**a–c**) show representative experiments, Panel (**d**) shows the data from three independent experiments.

Figure 4a shows the emission spectrum of ATA in the absence and presence of PMCA plus Ca^2+^ and Mg^2+^. Under these conditions, the conformational equilibrium of PMCA is shifted towards the *E*1Ca state. The ATA fluorescence increased in the presence of PMCA, and the λ_em_ maximum shifted slightly towards blue (from 425 to 429 nm) indicating that the ATA is in a more hydrophobic environment^11^. The fluorescence of ATA bound to PMCA increases as a function of ATA concentration with a saturable behavior (Fig. 4b), whereas in the absence of PMCA (free ATA) fluorescence increases linearly (*inset* in Fig. 4b). The continuous line corresponds to the fit of Eq. (3) to the experimental data,

3 $${F}_{ATA-PMCA}= \frac{{F}_{max}\cdot \left[ATA\right]}{{K}^{\prime}_{ATA}+ \left[ATA\right]},$$

where *Kʹ*_*ATA*_ corresponds to the ATA concentration at which half the maximum effect is achieved. The value obtained for *Kʹ*_*ATA*_ was 0.16 ± 0.02 µM. These results suggest that, under equilibrium conditions, only one molecule of ATA binds to PMCA with high affinity.

#### Intrinsic fluorescence of PMCA in the presence of ATA

The intrinsic fluorescence of PMCA is due to the presence of aromatic amino acids (Tyr, Phe, and Trp). Among these, Trp have the highest quantum yield, are very sensitive to environmental changes, and are the most susceptible to energy transfer (quenching) by charged residues or other ligands^24^. Figure 4c (*inset*) shows the excitation and emission spectra of PMCA and ATA. The emission spectrum of PMCA (*red line*) overlaps with the excitation spectrum of ATA (*green line*), indicating that the energy transfer from PMCA to ATA is possible. Furthermore, the emission spectrum of ATA (*dashed gray line*) does not overlap with that of PMCA, ensuring that the fluorescence at 332 nm arises solely from the intrinsic fluorescence of the protein. When increasing concentrations of ATA were added to PMCA and the samples were excited at 290 nm, a decrease in PMCA fluorescence concomitant with an increase in ATA emission was observed (Fig. 4c). Under these conditions, the main contribution of PMCA fluorescence is due to the Trp residues^25^.

Figure 4d shows that the PMCA fluorescence at 332 nm as a function of the ATA concentration decreased and then remained constant. Equation (4) was fitted to the experimental data,

4 $${F}_{PMCA}= {F}_{min}+\frac{{(F}_{0}-{F}_{min})\cdot {K}_{ATA}^{{\prime}{\prime}}}{{K}_{ATA}^{{\prime\prime}}+ \left[ATA\right]},$$

where *F*_0_ and *F*_*min*_ correspond to the intrinsic fluorescence of PMCA in the absence of ATA and when its concentrations tend to infinite, respectively, and *Kʹʹ*_*ATA*_ is the ATA concentration at which half the maximum effect is achieved. The values obtained for *F*_*min*_ and *Kʹʹ*_*ATA*_ were 0.69 ± 0.09 and 0.19 ± 0.02 µM, respectively. The value of *Kʹʹ*_*ATA*_ was not significantly different from *Kʹ*_*ATA*_ (from Eq. (3)) indicating that it is the same phenomenon observed from the changes that occur in PMCA (Fig. 4d) or in the ligand (Fig. 4b) when the PMCA·ATA complex is formed. Note, if we consider the approximation that most of ATA is in the free form ([ATA]_*free*_ ⁓ [ATA]_*TOTAL*_), the value of *Kʹʹ*_*ATA*_ (and *Kʹ*_*ATA*_) corresponds to the value of the apparent dissociation constant (*K*_*D*_) for the PMCA·ATA complex.

The representation of *F*_*0*_/*F* as a function of the quencher concentration is known as the Stern–Volmer plot and provides information about the interaction between a fluorophore and a quencher. Here, *F*_*0*_ and *F* represent the fluorescence in the absence and presence of a given quencher concentration ([Q]), respectively. The Stern–Volmer relationship assumes interaction between a single fluorophore and a quencher, proposing a linear relationship between *F*_0_/*F* and [Q]. Consequently, if PMCA had a single Trp residue (fluorophore) near the ATA-binding site, an increase in ATA concentration (quencher) would lead to a proportional decrease in the intrinsic fluorescence of the protein, resulting in a linear Stern–Volmer relationship. However, when multiple fluorophore molecules exist, such as in proteins with several Trp, the quencher may have varying degrees of accessibility to these residues, causing deviation from linearity in the Stern–Volmer plot with a negative curvature^24^. In the presence of saturating ATA concentrations, the intrinsic fluorescence of PMCA decreased by 30%, indicating that only a reduced group of Trp is close to the ATA-binding site and is accessible to quenching. The remaining intrinsic fluorescence (*F*_*min*_) is attributed to the residues distant from the ATA-binding site and thus, not accessible to quenching. The Stern–Volmer plot for PMCA and ATA deviates from linearity with a negative curvature (*inset* in Fig. 4d), suggesting the presence of more than one Trp near the ATA-binding site, each with different accessibility to quenching by ATA.

#### Effect of ATA on the nucleotide-binding pocket of PMCA

It has been proposed that ATA binds to the nucleotide-binding site of enzymes and inhibit their ATPase activity^6^. However, our results show that in PMCA, ATA behaves as a mixed-type inhibitor with respect to ATP, i.e. ATA does not bind to the ATP-binding site but its binding decreases the affinity of PMCA for this substrate. Thus, to evaluate whether ATA binds the ATP-binding site of PMCA in equilibrium conditions, we assayed the fluorescence of the PMCA∙ATA complex in the absence of Ca^2+^ (*E*2 state) to prevent the pump from cycling and in the presence of increasing ATP concentrations.

**Figure 5:**
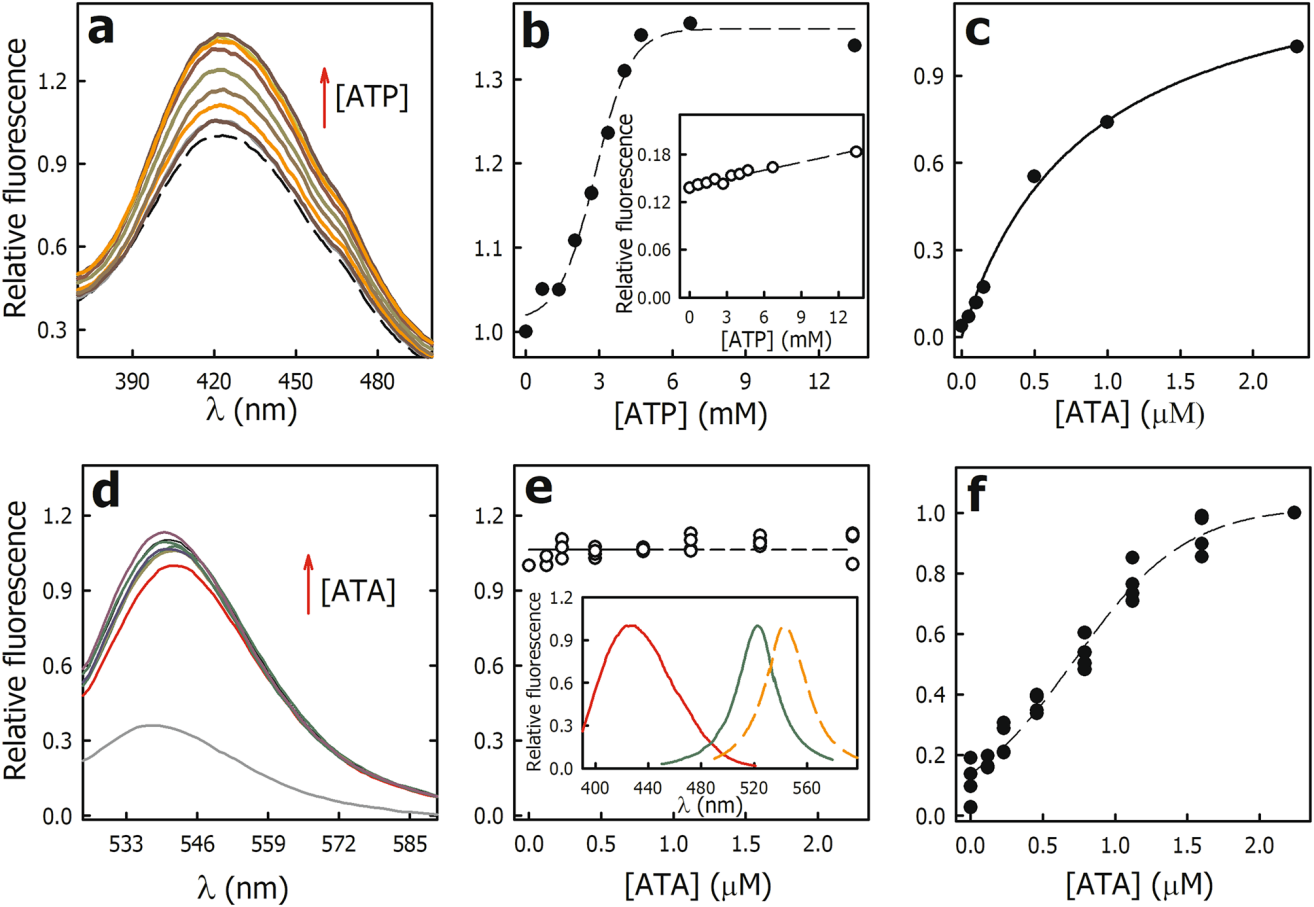
Effect of ATA on the nucleotide-binding pocket of PMCA. (**a**) Relative fluorescence of ATA bound to PMCA (PMCA·ATA complex) in the absence (*dashed black line*) and presence of increasing concentrations of ATP. (**b**) Relative fluorescence of ATA bound to PMCA (429 nm) as a function of ATP concentration. The dashed line corresponds to the fitting of Eq. (6) to the experimental data (see “Methods” section for details). *Inset*. Relative fluorescence of ATA (429 nm) as a function of ATP concentration in the absence of PMCA. In A and B, the fluorescence of ATA bound to PMCA at 429 nm in the absence of ATP was normalized to 1. (**c**) Relative fluorescence of ATA bound to PMCA (429 nm) in *E*2·ATP state. The continuous line represents the fitting of Eq. (3) to the experimental data and *Kʹ*_*ATA*_ was 0.9 ± 0.1 µM. (**d**) Relative fluorescence of Eo bound to PMCA (PMCA·Eo complex) in the absence (*red line)* and presence of increasing ATA concentrations. The gray line shows the fluorescence of Eo in the absence of PMCA. The fluorescence of Eo bound to PMCA at 543 nm in the absence of ATA was normalized to 1. (**e**) Relative fluorescence of Eo bound to PMCA (543 nm) as a function of ATA concentration. *Inset*. Emission spectra of ATA (*red line*) and the Eo (*dashed red line*), and excitation spectrum of Eo (*green line*). (**f**) Relative fluorescence of ATA bound to the PMCA·Eo complex (429 nm) as a function of ATA concentration. The dashed line corresponds to the fitting of Eq. (6) to the experimental data. In Panels (**c,f**), the fluorescence at 2 µM ATA was normalized to 1. Panels (**a–d**) show representative experiments, Panels (**e,f**) show the data from three independent experiments.

If ATP displaces ATA, the fluorescence of the PMCA∙ATA complex should decrease until values close to free ATA. On the contrary, the addition of ATP (ATP·Mg) produced an increase in the fluorescence, although without significant changes in the emission spectrum (Fig. 5a). Figure 5b shows that the fluorescence of the PMCA∙ATA complex at 429 nm increased with a saturable behavior (*dotted line*) as a function of ATP concentration, indicating that the binding of ATP to PMCA·ATA complex produces changes in the ATA environment. In the absence of PMCA, the addition of ATP produced a slight and linear increase in fluorescence (*inset* in Fig. 5b), consistent with its poor quantum yield under these experimental conditions^26^. Half of the maximum effect on the fluorescence was observed at 2.8 ± 0.1 mM ATP (Eq. 6), this value is two orders of magnitude greater than the dissociation constant for ATP in the *E*2 state of PMCA previously reported^21^. These results indicate that ATP binds to the PMCA∙ATA complex with a lower affinity than that to the free enzyme.

In another set of experiments, PMCA was incubated in the presence of 1 mM ATP and in the absence of Ca^2+^ (*E*2·ATP state). Then, increasing concentrations of ATA were added, and the fluorescence of the PMCA·ATA complex was determined (Fig. 5c). In the absence of ATA, the fluorescence of the PMCA·ATP complex was close to zero, and it increased as the concentration of ATA increased, indicating that ATA binds to the PMCA·ATP complex. The value of *Kʹ*_*ATA*_ obtained from the fitting of Eq. (3) to the experimental data was 0.8 ± 0.1 µM, a value significantly higher than that obtained in the absence of ATP (Fig. 4b). These results show that ATP binds to the PMCA·ATA complex and induces a change in the environment of ATA, affecting its fluorescence (Fig. 4b). Since ATP has a low quantum yield, we used the fluorescent probe eosin to evaluate whether ATA induces changes in the ATP-binding site environment.

Eosin (Eo) is a fluorescent probe that binds with high affinity (0.1 µM) to the ATP-binding site of PMCA inhibiting the Ca^2+^-ATPase activity by a competitive mechanism^27^. Eosin fluorescence is sensitive to changes in the hydrophobicity of its environment; thus, it can sense conformational changes that affect the nucleotide-binding pocket in the PMCA·Eo complex. When Eo binds to PMCA, its fluorescence increases and the emission maximum shifts from 539 to 543 nm. Consequently, the emission and excitation spectra of ATA and Eo do not overlap (*inset* in Fig. 5e), allowing us to specifically evaluate the fluorescence of ATA or Eo in the PMCA·Eo·ATA complex. Furthermore, the effect of ATA on the nucleotide-binding pocket can be studied in the presence of Ca^2+^ (*E*1Ca). To evaluate whether ATA displaces Eo from the ATP-binding site of PMCA, the PMCA·Eo complex was formed, and increasing concentrations of ATA were added. Then, the fluorescence at 543 nm (PMCA·Eo complex) and at 423 nm (PMCA·ATA complex) was determined in each sample.

Figure 5d shows the emission spectrum of Eo in the absence (*gray line*) and the presence of PMCA and Ca^2+^ (PMCA·Eo complex, *red line*). The addition of ATA to the PMCA·Eo complex produced a slight increase in the fluorescence and a slight redshift of the emission maximum, suggesting that the environment of Eo in the nucleotide-binding pocket changes. The fluorescence of the PMCA·Eo complex at 543 nm as a function of ATA concentration remained constant (Fig. 5e), indicating that ATA does not displace Eo from the nucleotide-binding pocket. In the same samples, the fluorescence of the PMCA·ATA complex increased with a saturable behavior (Fig. 5f), suggesting that ATA binds to the PMCA·Eo complex. Half of the maximum effect on the fluorescence was observed at 0.72 ± 0.01 µM ATA (Eq. 6), a value higher than that obtained in the absence of Eo (Fig. 4b). Taken together, these results indicate that ATA can bind to the PMCA·ATP complex (and to PMCA·Eo) but with a lower affinity than to the free enzyme. Consistent with this, ATP also binds to the PMCA·ATA complex with lower affinity than to free PMCA.

#### The ATA-binding site assayed by flexible molecular docking

To find a possible binding site of ATA in PMCA, we performed flexible molecular docking studies. From this point forward, we will denote the immediate environment surrounding the ATP-binding site as nucleotide-binding pocket.

##### The ATP-binding site

**Figure 6:**
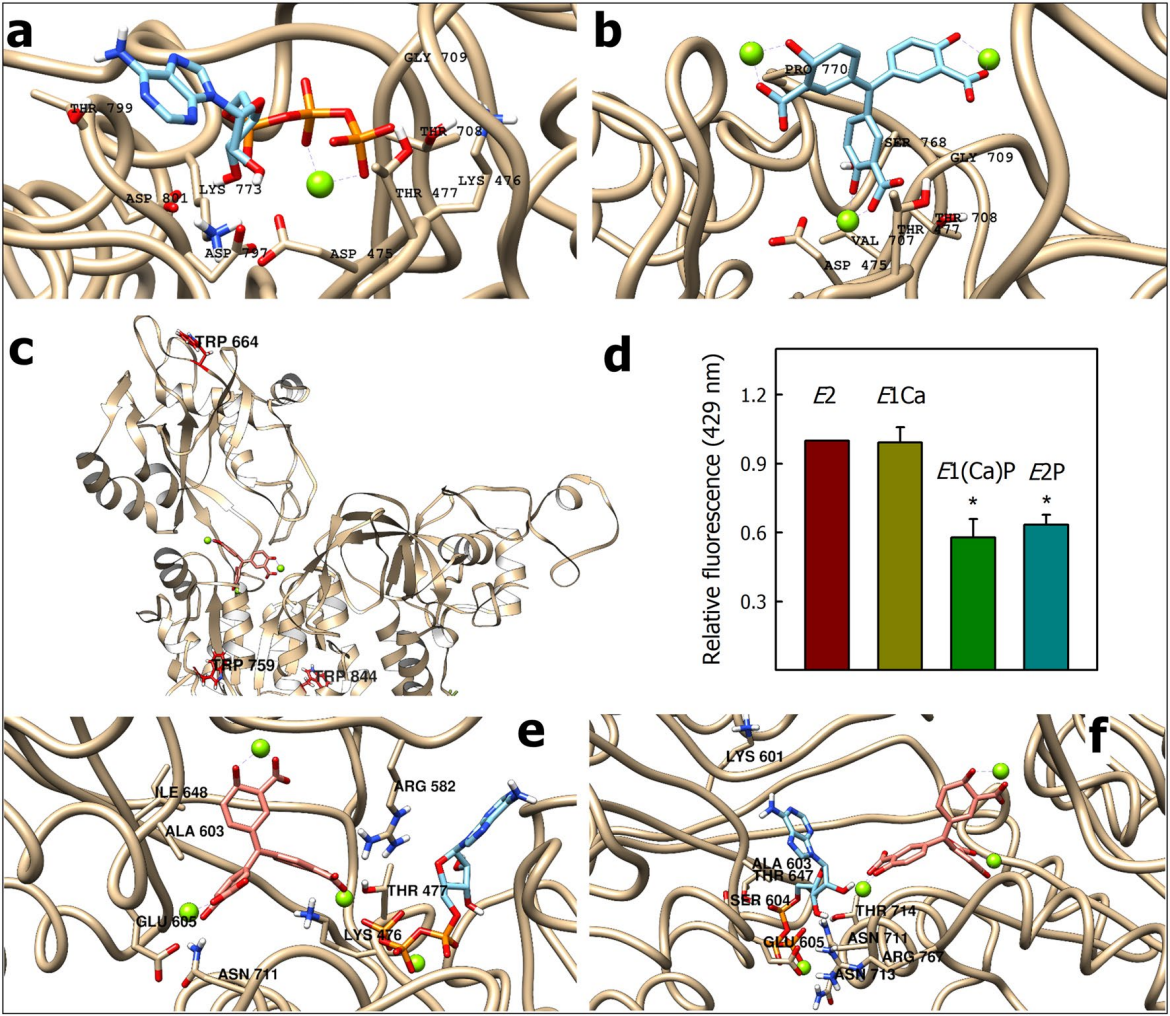
The ATA-binding site. (**a**) Propose ATP-binding site of PMCA. All residues identified in PMCA1 are conserved in PMCA4 as follow (PMCA1 → PMCA4): Asp475–Lys476–Thr477 → Asp465–Lys466–Thr467; Thr708–Gly709 → Thr696–Gly697; Lys773 → Lys661; Asp797–Trh799–Asp801 → Asp785–Trh787–Asp789. (**b**) Propose ATA·Mg-binding site on the PMCA1 structure. All residues identified in PMCA1 are conserved in PMCA4 as follow (PMCA1 → PMCA4): Asp475–Thr477 → Asp465–Thr467; Val707–Thr708–Gly709 → Val695–Thr696–Gly697; SER768–Pro770 → SER756–Pro758. (**c**) Trp residues of PMCA near ATA·Mg: Trp759 (P-domain) at 20.8 Å, Trp844 (P-domain) at 29.9 Å and Trp664 (N-domain) at 34.5 Å. These residues correspond to Trp 747, Trp 832 and Trp652 in PMCA4. (**d**) Relative fluorescence of ATA bound to *E*2, *E*1Ca, *E*1(Ca)P and *E*2P (*E*2·BeF) states of PMCA. The fluorescence of ATA bound to *E*2 state was normalized to 1. (**e**) Binding of ATA·Mg to PMCA·ATP complex. (**f**) Binding of PMCA·ATP to ATA·Mg complex. Color code: The oxygen, carbon, phosphorus and hydrogen atoms are colored red, blue, orange and white, respectively. The magnesium ion is represented as a green sphere. The ATA molecule is shown in blue light (**b**) or pink (**c,e,f**). In (**d**), the values are the mean ± SE of three independent experiments. *P < 0.01 with respect to *E*2.

Because PMCA structure has not been resolved in the presence of ATP, we first performed molecular docking of ATP (ATP·Mg) on PMCA1. Figure 6a shows the predicted ATP-binding site of PMCA1 in the absence of Ca^2+^ (*E*2). As residues interacting with ATP are conserved in P-ATPases, we compared the results obtained by molecular docking in PMCA1 (first position =  − 9.3 kcal/mol) with residues identified in the *E*2·ATP structure of SERCA1^28,29^ (detailed in parentheses in the text). All residues identified in PMCA1 are conserved in PMCA4 (see legend of Fig. 6a). In PMCA, the γ-phosphate interacts with Lys773 (Lys684^30^) and Lys476 through salt bridges, and with Thr708 (Thr 625), Gly709 (Gly626), and Thr477 (Thr353) through hydrogen bonds. Lys476 and Thr477 belong to the 475DKTG motif, which contains the phosphorylatable Asp475, and Thr708 and Gly709 belong to the 708TGDN motif^31^. Nearby, Asp475 (Asp351) and Asp797 (Asp703) interact through the Mg^2+^ ion^29^, Thr799 (Val705), and Asp801 stabilize the nucleotide through hydrogen bonds. Residues Asp797, Thr799, and Asp801 belong to the 795TGDVND motif (701TGDXND), which together with the 708TGDN motif, is involved in the coordination of Mg^2+^ upon the binding of ATP^32^. This suggests that the predicted site may be the ATP-binding site in PMCA.

##### The ATA-binding site

In the molecular docking of ATA uncomplexed with Mg (ATA(−)) all predicted conformations overlap with the ATP-binding site. In the most probable conformation (− 8.3 kcal/mol), all residues that interact with ATA(−) coincide with those proposed for the interaction with ATP (Supplementary Fig. S1). Therefore, the proposed ATA(−) binding site on PMCA does not match experimental results showing that this ligand does not bind to the ATP-binding site. In the molecular docking of ATA·Mg, all predicted conformations are possible and are close to the ATP-binding site (Supplementary Fig. S2). Figure 6b shows the ATA·Mg-binding site in the most likely conformation (− 9.6 kcal/mol). ATA·Mg interacts through Mg^2+^ ions with Asp475 and Val707 and, establishes hydrogen bonds with Thr477, Thr708, and Gly709. Thus, ATA·Mg would interact with residues of the 708TGDN and 475DKTG motifs, involved in the interaction with Mg^2+^ and the γ-phosphate of ATP at the phosphorylation site (Asp475)^32^. Furthermore, ATA·Mg interacts with Ser768 through a hydrogen bond and is stabilized by hydrophobic interactions with Pro770. These latter residues are in the nucleotide-binding pocket but are not involved in the interaction with ATP (Fig. 6a). Unlike ATA(−), ATA·Mg does not interact with Lys773 and Lys476, two key residues for the interaction with ATP^31^. A similar result has been described in the serine/threonine phosphatase Stp1^4^, where ATA binds into the active pocket inhibiting the phosphatase activity by a mixed-type mechanism. On the other hand, three of the ten Trp residues of PMCA are located less than 35 Å apart from ATA-Mg: Trp759 (P-domain) at 20.8 Å, Trp844 (P-domain) at 29.9 Å and Trp664 (N-domain) at 34.5 Å (Fig. 6c). The other Trp are located at the interface with the transmembrane domain, at a distance greater than 35 Å from the ATA·Mg. This agrees with the fact that only a small fraction of the Trp of PMCA is differentially accessible to quenching by ATA (Fig. 4d). All residues mentioned for the ATA·Mg interaction in PMCA1 are conserved in PMCA4 (see legend of Fig. 6b).

The nucleotide-binding pocket of PMCA is less accessible to water in the *E*1Ca and *E*2 states than in the *E*P analogous states^14,27^. These changes in the hydrophobicity affect the fluorescence of probes that bind in the nucleotide-binding pocket. The quantum yield of Eo is higher in hydrophobic environments; thus, its fluorescence is higher when it is bound to the *E*1Ca and *E*2 states than to the *E*2P and *E*1(Ca)P states. Since the molecular docking results propose that ATA·Mg binds in the nucleotide-binding pocket, we evaluated the fluorescence of ATA bound to PMCA in the states analogous to *E*2P and *E*1(Ca)P. To this end, we phosphorylated PMCA from ATP in the presence of Ca^2+^ and lanthanum to stabilize the *E*1(Ca)P state, or stabilized the *E*2P-analogous form in the absence of Ca^2+^ and the presence of BeF_3_^−^ (see “Methods” section for details). Then, we determined the fluorescence of the PMCA⋅ATA complex. Similar to that occurs with Eo^14,27^, the fluorescence of ATA bound to PMCA in the *E*1Ca and *E*2 states was similar, while when the pump was stabilized in the states analogous to *E*2P and *E*1(Ca)P, the fluorescence decreased (Fig. 6d). Consistent with the site proposed by flexible molecular docking (Fig. 6b), these results show that ATA would be sensitive to changes in the nucleotide-binding pocket of PMCA.

##### The binding of ATP and ATA to PMCA

While the flexible molecular docking results indicate a partial overlap between the ATA·Mg-binding site and the ATP-binding site, experimental evidence suggests that this interaction does not follow a competitive inhibition mechanism, i.e. ATA and ATP bind simultaneously to PMCA. To test this, we assayed whether ATA·Mg and ATP could bind both to the PMCA nucleotide-binding pocket. Figure 6e shows the molecular docking of ATA·Mg to the PMCA·ATP complex in the most probable conformation (Fig. 6a). The ten predicted conformations are feasible and are near the ATP-binding site, but the binding energy of ATA·Mg increases from − 9.8 to − 8.7 kcal/mol. In the presence of ATP, ATA·Mg maintains its interaction with Thr477 and interacts with Glu605 through the Mg^2+^ ion. It is further stabilized by hydrogen bonds with Lys476 and Asn711 (part of the 708TGDN motif) and by a salt bridge with Arg582. Notably, Arg582 is homologous to Arg489 in SERCA and stabilizes the γ-phosphate of the ATP analog adenosine 5′-[α,β-methylene]diphosphate (AMPPCP) in the *E*2-AMPPCP state^31^. Figure 6f shows the molecular docking of ATP to the PMCA·ATA·Mg complex in the most probable conformation (Fig. 6b). Only four predicted conformations are feasible and the binding energy of ATA·Mg increases from − 9.3 to − 8.4 kcal/mol in the most probable position. ATP binds near the ATA·Mg, where it is stabilized by hydrogen bonds with Asn711, Asn713, Thr714, and Glu605, among other residues. Although ATA·Mg is not coordinated by residues of the 795TGDXND motif, the γ-phosphate of ATP loses interaction with these residues and is coordinated by others in the nucleotide-binding pocket. Furthermore, in the PMCA·ATA·Mg complex, ATP would lose interaction with Lys773 and the 475DKTG motif. It is important to note that in these experiments the molecular docking of ATA·Mg or ATP on the PMCA-ATP or PMCA·ATA·Mg complex is conducted, respectively. Consequently, the ligand already bound within the complex doesn’t exhibit free movement, as would be the case, given the reversible binding nature of both molecules to PMCA. Thus, the primary objective of these experiments is to assess the compatibility of both ligands within the nucleotide-binding pocket. However, the interaction with different residues must be analyzed with prudence.

These findings suggest that while the ATA·Mg binding site partially overlaps with the ATP binding site, both ligands can accommodate within the nucleotide-binding pocket, albeit with lower binding affinity compared to free PMCA.

## Conclusions

In PMCA, ATA behaves as a mixed-type inhibitor with respect to Ca^2+^ and ATP, and as an uncompetitive inhibitor with respect to Mg^2+^. Although these models should be interpreted with caution within the framework of P-ATPase complexity, they show that Mg^2+^ favors inhibition and rule out ATA behaving as a competitive inhibitor with respect to ATP. In equilibrium conditions, fluorescence studies show that ATA binds to the *E*1Ca and *E*2 states of PMCA with similar affinity in the submicromolar range. Consistent with a mixed-type inhibition mechanism, ATP binds to the PMCA·ATA complex and ATA binds to the *E*2·ATP state of PMCA, in both cases with lower affinity than to the *E*2 state (free enzyme). The same occurs in the presence of Ca^2+^, where the state analogous to *E*1Ca·ATP is evaluated with the fluorescent probe Eo bound to the ATP-binding site.

To explore a potential ATA-binding site, flexible molecular docking studies were conducted. Since ATA(−) has been used in predicting and analyzing experiments involving other ATP-binding proteins^4,7,8^, the binding of both ATA·Mg and ATA(−) was assessed. The findings revealed that the ATA(−)-binding site overlaps with the ATP-binding site, whereas ATA·Mg is located within the nucleotide-binding pocket, where it interacts with residues of the phosphorylation and Mg^2+^ interaction motifs. Consistent with this, ATA binds to the *E*1(Ca)P and *E*2P states of PMCA and respond to the hydrophobicity of the nucleotide-binding pocket environment. There, only three Trp residues (Trp759 in the N-domain, and Trp844 and Trp664 in the P-domain) are less than 35 Å from ATA·Mg, consistent with the observed partial quenching of the PMCA intrinsic fluorescence. Furthermore, molecular docking experiments also predict that ATA·Mg and ATP can fit together in the nucleotide-binding pocket, although with higher binding energy when the other ligand is present. On the other hand, although the interaction of ATA with divalent and trivalent cations has been widely documented, this aspect has not been considered in most studies involving its interaction with proteins. As our results demonstrate, this is a crucial point to consider in experiment design and interpretation.

In summary, this study establishes a robust groundwork for future research endeavors, focusing on elucidating the interaction between ATA and proteins, as well as advancing the identification of inhibitors for the development of pharmacological strategies to mitigate disorders linked to PMCA dysfunction.

## Methods

### Reagents

All chemicals used in this work were of analytical grade and purchased from Sigma-Aldrich and Merck. For the isolation of PMCA, we used recently donated, safe (tested) human blood, obtained from the Hematology Section of the *Fundosol Foundation* (Argentina), shortly after reaching the shelf life (expiration date) for the use in humans. Donating blood in Argentina is voluntary and, therefore, the donor gives his informed consent for the donation of blood and for the subsequent legitimate use of the blood by the transfusion service. *Fundosol Foundation* performs all procedures following the ethical and safety protocols approved for blood donation in Argentina (law 22.990). The purification of PMCA from red blood cells was carried out following standard safety protocols approved by IQUIFIB.

### Purification of PMCA from human erythrocytes

PMCA was isolated from calmodulin-depleted human erythrocyte membranes (90% PMCA4b and 10% PMCA1)^15^ by affinity chromatography in a calmodulin-agarose column as described previously with some modifications^33^. Purified PMCA was reconstituted in buffer A, containing 20% (m/v) glycerol, 72 μg/ml C_12_E_10_, 24 μg/ml DMPC, 120 mM KCl, 2 mM MgCl_2_, 30 mM MOPS-K (pH 7.4 at 4 °C), 2 mM EGTA, 2 mM dithiothreitol (DTT) and stored under liquid nitrogen until use. Protein concentration after purification was about 25 μg/ml.

### Measurement of Ca^2+^‐ATPase activity

Ca^2+^‐ATPase activity was determined at 37 °C from the slope of the curve of inorganic phosphate (P_i_) release from ATP as a function of time. The amount of Pi was determined by the Fiske and Subbarrow method as described previously by Saffioti et al.^14^. In each condition, the amount of Pi released in the absence of Ca^2+^ was subtracted from the P_i_ released in its presence. The experimental setup was adjusted to ensure that PMCA initial velocity conditions were met. Except when specified in the text, the reaction medium contained 10 nM PMCA, 120 mM KCl, 30 mM MOPS-K (pH 7.2 at 37 °C), 120 μM C_12_E_10_, 35 μM DMPC, 2 mM MgCl_2_, 2 mM EGTA 2.1 mM CaCl_2_ (final free Ca^2+^ concentration of 80 μM). In this condition, PMCA Ca^2+^-ATPase activity is optimal^34^. The reaction was started by the addition of ATP. Free Ca^2+^ and Mg^2+^ concentrations were calculated in the presence of 2 mM EGTA, 2 mM ATP, 37 °C, ionic strength 0.12 and pH 7.4 with MAXCHELATOR web program^35^. When one ligand (Ca^2+^, Mg^2+^ or ATP) was variable the other two were constant at their optimal concentration (indicated above). In Fig. 2, Eq. (5) was fitted to experimental data.

5 $$v=\frac{{V}_{max}[x]}{{K}_{x}+[x]},$$

where [*X*] represents the Ca^2+^ (a), Mg^2+^ (b) o ATP (c) concentration, *V*_*max*_ is the Ca^2+^-ATPase activity when [*X*] tends to infinity and, *K*_*x*_ (*K*_*Ca*_*; K*_*Mg*_* or K*_*ATP*_*)* is the X concentration at which the halfmaximum effect is achieved. ATA was dissolved in ethanol in 100× concentration and stayed sheltered from the light.

### Measurement of phosphatase activity

Phosphatase activity was determined from the slope of the curve of *p*‑nitrophenol release from *p*-nitrophenyl phosphate (pNPP) as a function of time. Assays were carried out at 37 °C as described previously by Mazzitelli and Adamo^19^. The experimental setup was adjusted to ensure that PMCA initial velocity conditions were met. The reaction medium contained 15 nM PMCA, 120 mM KCl, 10 mM MgCl_2_, 120 μM C_12_E_10_, 35 μM DMPC, 2 mM EGTA and 30 mM MOPS-K (pH 7.2 at 37 °C). The reaction was initiated by the addition of 5 mM p-nitrophenyl phosphate (pNPP) and stopped with 1 ml of 0.1N of NaOH. The amount of pNPP was determined by its Abs (410) using its molar absorptivity constant (1.78 × 104 M^−1^ cm^−1^) after subtracting the Abs (410) in the absence of PMCA (blank).

### Measurement of fluorescence

Fluorescence measurements were assessed in a Jasco FP-6500 fluorometer, in a 3 × 3 mm quartz cuvette, and at 25 °C with a Peltier-controlled temperature. Fluorescence was expressed relative to a condition (Relative Units). When consecutive volumes of probe or reagent were added, the final dilution did not exceed 10%.

### Fluorescence of ATA

The excitation (λ_ex_) and emission (λ_em_) wavelengths of ATA were 306 nm and 429 nm, respectively. In all experiments, PMCA (50 nM) was reconstituted in the presence of 120 mM KCl, 30 mM MOPS-K (pH7.3 at 25 °C), 120 μM C⁠_12_E_⁠10_, 35 μM DMPC, 20% glycerol and 2 mM MgCl⁠_2_. When is indicated, the binding of ATA to PMCA was evaluated in the presence of: 2 mM EGTA (*E*2 state); 80 μM free Ca^2+^ (*E*1Ca state); 1 mM ATP (*E*2·ATP)^21^; 80 μM free Ca^2+^, 100 μM LaCl_3_, 25 μM ATP (*E*1(Ca)La (analog to *E*1(Ca)P^36^ or 40 μM BeCl_2_, 2 mM NaF (*E*2BeF, analog to *E*2P^27^. In each condition assayed, the fluorescence of ATA in an identical solution but in the absence of protein (free ATA) was subtracted to obtain the fluorescence of ATA bound to PMCA (PMCA·ATA complex)^14,27^. In Fig. 5a,b, PMCA·ATA complex was formed in the presence of 0.4 μM ATA. In Fig. 5b,f, the empirical Eq. (6) was fitting to experimental data to obtain the concentration of ATP or ATA at which half the maximum effect on the fluorescence of the PMCA·ATA (b) and PMCA·Eo (f) complexes was achieved.

6 $$y={y}_{0}+ \frac{a}{1+{e}^{-\left(\frac{x-{x}_{0}}{b}\right)}}.$$

### Fluorescence of eosin

The λ_ex_ and λ_em_ of Eosin (Eo) were 520 nm and 543, respectively. In Fig. 5, Eo bound to PMCA (PMCA·Eo complex) was assayed in the presence of 80 μM free Ca^2+^ (*E*1Ca) and 0.3 μM Eo as described previously by Saffioti et al.^27^.

### Intrinsic fluorescence of PMCA

When the intrinsic fluorescence of PMCA was evaluated, the sample was treated as described above (*E*2 state). The λ_ex_ and λ_em_ used were 290 nm and 332 nm, respectively.

### Flexible molecular docking studies

The structure of the plasma membrane calcium pump PMCA1 was retrieved from the Protein Data Bank (PDB) repository, specifically from the cryo-EM structure solved with a resolution of 4.1 Å (PDB ID: 6A69)^16^. The sequence alignment between PMCA1; PMCA4 and/or SERCA1 was performed with BLAST (https://blast.ncbi.nlm.nih.gov/)^37^. The structure of the ligand Triaurinecarboxylic Acid (ATA(−)) was generated using Avogadro software^38^, facilitating molecule editing and visualization. Hydrogens were added based on a pH of 7.4, followed by geometric optimization of the molecule. To construct the structure of the ligand Triaurinecarboxylic Acid coordinated with Magnesium (ATA·Mg), calcium atoms from the structure loaded in the open chemistry database PubChem^39^ were coordinated and replaced by magnesium atoms. Based on the structure of SERCA in the presence of the ATP analogue AMPPCP (PDB code 1VFP^28^), the ATP·Mg molecule corresponds to the bidentate conformation, where the Mg^2+^ ion is coordinated by the β and γ phosphate groups^40^ and was taken from the PDB I.D: 1XEF^41^. Molecular docking analysis was performed utilizing AutoDock Vina 1.1.2 software integrated into UCSF Chimera 1.16 (http://www.rbvi.ucsf.edu/chimera)^42^. The preparation of the system involved the elimination of water molecules, addition of hydrogen atoms at a pH of 7.4, assignment of Gasteiger charges, and the retention of default parameters. Adjustments to Grid Box coordinates and box size were made according to the volume of the binding site, with a maximum of ten resulting conformations specified. We considered “possible conformations” to be those in which the ligand exhibited a binding energy lower than − 7.5 kcal/mol. The type and number of contacts established by each ligand conformation (ATA(−), ATP, or ATA·Mg) with PMCA were determined using the web service Protein Ligand Interaction Profiler (PliP)^43^. Each proposed conformation was simulated using molecular dynamics with GROMACS^44^, following standard protocols, to assess the stability of the ligand at the site.

### Data analysis and simulated curves

Theoretical equations were fitted to the results by nonlinear regression based on the Gauss–Newton algorithm using commercial programs (Excel and Sigma-Plot for Windows, the latter providing not only the best fitting values of the parameters but also their standard errors). The goodness of fit of a given equation to the experimental results was evaluated by the corrected AIC (Akaike Information Criterion)^45^ defined as AIC C = N ln(SS/N) + 2P N/(N − P − 1), where N is the number of data, P is the number of parameters plus one, and SS is the sum of weighted square residual errors. In Fig. 6, the value of P was calculated from a two-tailed Student’s test.

### Supplementary Information

Supplementary Figures.

## Data Availability

All data supporting the results of this manuscript are found in the main text. In addition, results based on flexible molecular docking, which correspond to Fig. 5a–c,e, Supplementary Figs. S1, S2 and S3 are available in https://figshare.com/articles/figure/Souto-Guevera_y_cols_/25564485.

## Abbreviations

PMCA: Plasma membrane Ca^2+^-ATPase
ATA: Aurintricarboxylic acid
ATA·Mg: Aurintricarboxylic acid complexed with Mg^2+^
