# Supplementary material for: Magnesium enhances aurintricarboxylic acid’s inhibitory action on the plasma membrane Ca2+-ATPase
Source: Sci Rep. 2024 Jun 26;14:14693. doi: 10.1038/s41598-024-65465-8 (PMC11208427; doi:10.1038/s41598-024-65465-8)
Supplement: Supplementary file 1 — Supplementary Figures. [file 41598_2024_65465_MOESM1_ESM.pdf]

SUPPLEMENTARY INFORMATION

Magnesium Enhances Aurintricarboxylic Acid's Inhibitory Action on the plasma membrane Ca^{2+}-ATPase

Cecilia A. Souto-Guevara[1], Diego Obiol[2], Camila L. Bruno[1], Mariela S. Ferreira-Gomes[1], Juan Pablo F. C. Rossi[1], Marcelo D. Costabel[2] and Irene C. Mangialavori[1*]

[1]From Universidad de Buenos Aires, Consejo Nacional de Investigaciones Científicas y Técnicas (CONICET). Instituto de Química y Fisicoquímica Biológicas Dr. Alejandro Paladini (IQUIFIB), Facultad de Farmacia y Bioquímica, C1113AAD Ciudad Autónoma de Buenos Aires, Argentina.

[2]From Instituto de Física del Sur (IFISUR), Departamento de Física, Universidad Nacional del Sur (UNS), CONICET, B8000CPB Bahía Blanca, Argentina.

Figure S1: The ATA(-)-binding site in PMCA

The ATP-binding site is highly conserved in P-ATPases, particularly in PMCA and SERCA, whose structure has been resolved in various conformations. Based on this evidence, Figure S1 illustrates that the residues involved in the interaction with ATA(-) coincide with those proposed in Figure 6A for the ATP interaction. This suggests an overlap between the ATA(-)-binding site and the ATP-binding site in PMCA.

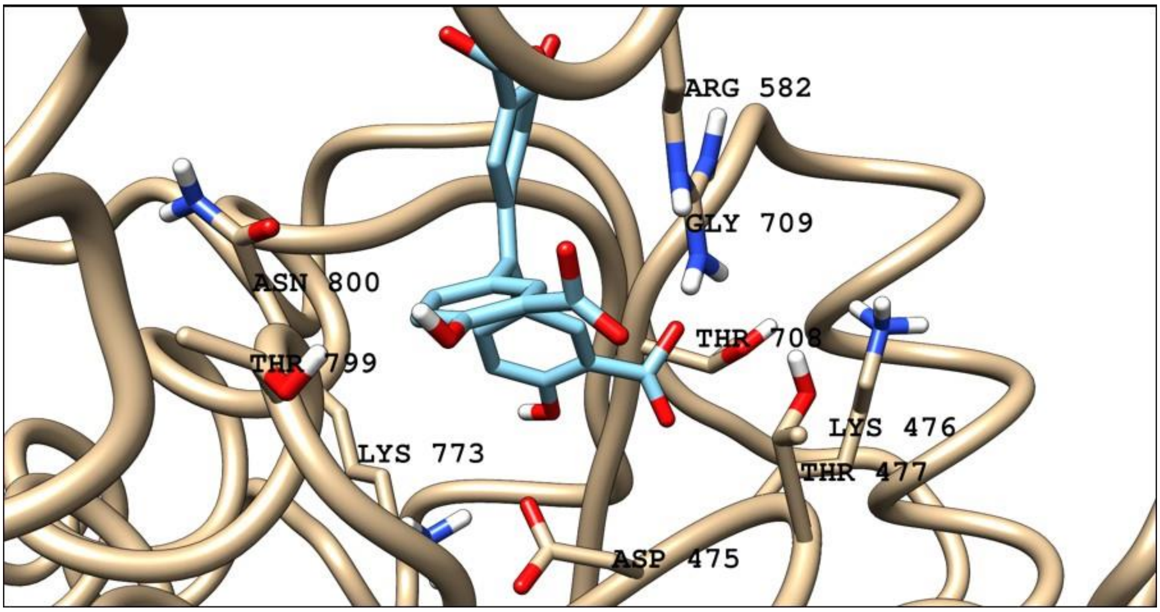

Figure S1: The ATA(-)-binding site on PMCA. The ATA(-) molécule is shown in blue ligth. The oxygen, carbon and hydrogen atoms are colored red, blue and white, respectively.

Figure S2: The ATA·Mg-binding site in PMCA

According to flexible molecular docking studies, the 10 possible conformations in which ATA·Mg can bind to PMCA occur in the same pocket (nucleotide-binding pocket). Considering the highest binding energy as the most likely interaction meanings 1 to 10, panels show: (A) poses 1, 2, 4, 5 and 6; (B) poses 3, 7 and 10, and, (C) poses 8 and 9.

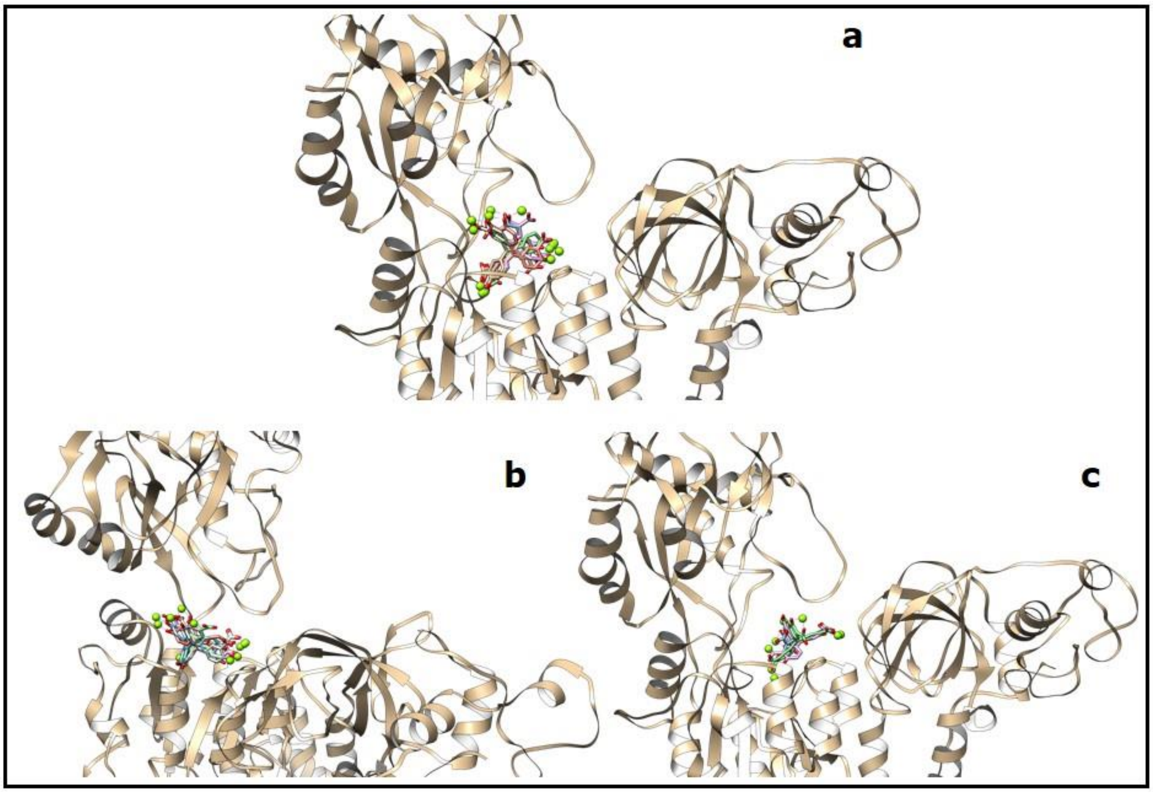

Figure S2: The ATA·Mg-binding site in PMCA. (A) poses 1 (-9.6 kcal/mol), 2 (-9.5 kcal/mol), 4 (-9.2 kcal/mol), 5 (-9.1 kcal/mol) and, 6 (-9.1 kcal/mol); **(B)** poses 3 (-9.3 kcal/mol), 7 (-9.0 kcal/mol) and, 10 (-8.9 kcal/mol), **(C)** poses 8 (-9.0 kcal/mol) and 9 (-8.9 kcal/mol). The oxygen atoms are colored red and the Mg^{2+} ions are represented as green spheres.

Figure S3: The binding of ATA·Mg to PMCA·ATP complex

In the molecular docking of ATA·Mg to the PMCA·ATP complex, the ten predicted conformations are feasible and they superpose near ATP-binding site. Respect to free PMCA, the binding energy of ATA·Mg to PMCA·ATP

increases from -9.8 to -8.7 kcal/mol. The ten poses have binding energy from -8.7 to -8.0 kcal/mol.

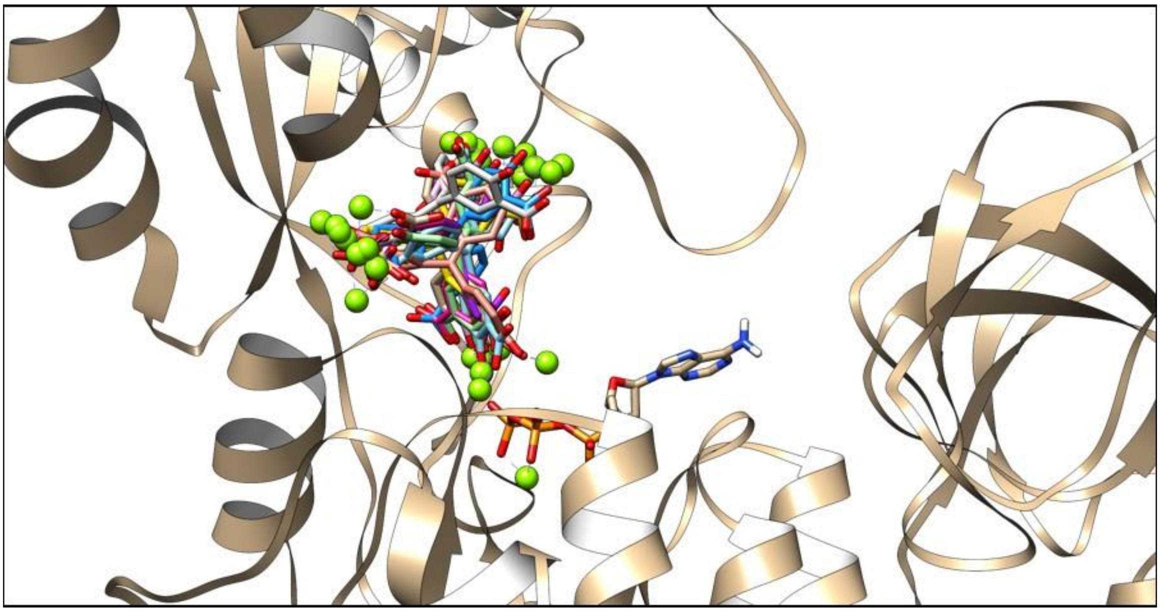

Figure S3: The binding of ATA·Mg to PMCA·ATP complex. The oxygen, carbon and hydrogen atoms are colored red, blue and white, respectively. The Mg^{2+} ions are represented as green spheres.
